# Long-term effects of malnutrition on severity of COVID-19

**DOI:** 10.1038/s41598-021-94138-z

**Published:** 2021-07-22

**Authors:** Alec Kurtz, Kenneth Grant, Rachel Marano, Antonio Arrieta, Kenneth Grant, William Feaster, Caroline Steele, Louis Ehwerhemuepha

**Affiliations:** 1 Albany Medical College, 43 New Scotland Avenue, Albany, NY 12208 USA; 2 Children’s Hospital of Orange County, 1201 W La Veta Ave, Orange, CA 92868 USA; 3 Schmid College of Science, Chapman University, 1 University Drive, Orange, CA 92866 USA

**Keywords:** Health care, Medical research, Risk factors, Diseases, Infectious diseases, Nutrition disorders

## Abstract

The COVID-19 pandemic is a public health crisis that has the potential to exacerbate worldwide malnutrition. This study examines whether patients with a history of malnutrition are predisposed to severe COVID-19. To do so, data on 103,099 COVID-19 inpatient encounters from 56 hospitals in the United States between March 2020 and June 2020 were retrieved from the Cerner COVID-19 Dataset. Patients with a history of malnutrition between 2015 and 2019 were identified, and a random intercept logistic regression models for pediatric and adult patients were built controlling for patient demographics, socioeconomic status, admission vital signs, and related comorbidities. Statistical interactions between malnutrition and patient age were significant in both the pediatric [log-odds and 95% confidence interval: 0.094 (0.012, 0.175)] and adult [− 0.014 (− 0.021, − 0.006] models. These interactions, together with the main effect terms of malnutrition and age, imply higher odds for severe COVID-19 for children between 6 and 17 years with history of malnutrition. Even higher odds of severe COVID-19 exist for adults (with history of malnutrition) between 18 and 79 years. These results indicate that the long-term effect of malnutrition predisposes patients to severe COVID-19 in an age-dependent way.

## Introduction

Balanced nutritional intake during the progression of and recovery from any illness is important for improvement in health outcomes^1–3^. Therefore, it is expected that malnutrition may have deleterious effects on the prognosis of the novel Coronavirus Disease 2019 (COVID-19) and therefore require proper attention^4–9^. The immediate nutritional risk that the COVID-19 pandemic created is the increased risk of malnutrition due to the economic impact of social distancing, full or partial lockdowns, and quarantining^10^. Many households have suffered loss of income and/or sources of supplemental food such as school meals for children. Although the causes of malnutrition are multifactorial, government-imposed shutdowns and quarantines have caused further shifts in the food industry and dietary practices^10,11^.

A more direct impact of COVID-19 on malnutrition has been observed. There have been increases in the incidence of malnutrition among older adults with severe COVID-19^12–14^. This suggests that COVID-19 or its complications may be triggering malnutrition or related physiological processes. This is of crucial importance because malnutrition dampens the immune system, increases mortality, hospital length of stays, and the risk of unplanned hospital readmission^15–17^. Furthermore, acute and chronic malnutrition predispose patients to significantly increased risk of bacterial and viral infection and increased severity of these infections^15^. Consequently, it is expected that a malnourished patient will have a worse prognosis than a patient without nutritional deficiency in all diseases as well as in severe COVID-19.

There is, however, the question of nutritional deficiencies not brought upon by COVID-19 or during hospitalization for COVID-19. Could there be a long-term effect of malnutrition and severity of COVID-19 in hospitalized patients? Does the associated risk (if any) depend on the age of the patient and is the mechanism essentially different between the pediatric and adult populations? The objective of this study is to assess whether patients with documented history of malnutrition are at higher risk of severe COVID-19 and compare the results between children and adults. In essence, does any degree of malnutrition predispose patients to severe COVID-19? This information may help with the development of proactive intervention protocols and revisions of existing nutritional care of patients hospitalized with COVID-19.

## Methods

The data source was the COVID-19 dataset from the Cerner® Real World Data (CRWD) which is fully de-identified database curated and managed by Cerner Corporation^18^. The June version of the COVID-19 subset of the CRWD for hospitalizations between March 1, 2020 and June 30, 2020 were retrieved and used for this retrospective case–control study.

### Patients and variables

COVID-19 hospitalization data were retrieved for both children and adults including patient and health insurance data, first vital signs during the encounter, and diagnoses data of patients between 2015 and 2019 prior to hospitalization with COVID-19. The health system each patient belongs to was also retrieved for statistical modeling purposes.

According to the American Society of Parenteral and Enteral Nutrition (ASPEN), malnutrition is a clinical condition that may be associated with weight loss over time, inadequate energy intake compared with estimated needs, muscle loss, fat loss, fluid accumulation, and diminished grip strength^19–21^. ASPEN defines pediatric malnutrition as an imbalance between intake and nutritional requirements resulting in cumulative energy, protein, or micronutrient deficits which may negatively affect growth and development^21^. Diagnosis of malnutrition is often undertaken through special assessments by a dietitian. The most reliable diagnosis of malnutrition is therefore in the assessments by dietitians which may result in the selection of the appropriate diagnosis code for the patient. We chose to use the diagnosis codes for malnutrition because it is available and the most practical approach with multicenter electronic medical records databases. The definitions of malnutrition have also evolved over the years and biomarkers such as serum albumin or other laboratory data have been identified as insufficient for proper diagnosis. The use of more stringent criteria is infeasible for large multicenter studies. Small multicenter studies with access to appropriate nutritional assessments and malnutrition screening tools are the gold standard for diagnosis of malnutrition. Lastly, we grouped all 3 levels of malnutrition (severe, moderate, and mild) into a single category to avoid extreme class imbalance and to estimate the average effect of malnutrition across all 3 levels. Therefore, patients with malnutrition were identified using the International Classification of Disease Version 10, Clinical Modification (ICD-10-CM) codes between E40 and E46 – these codes encompass severe, moderate, and mild malnutrition.

Comorbid conditions of the COVID-19 patients in this study were retrieved and classified by chapters of the ICD-10-CM codes. Only conditions diagnosed between 2015 and 2019 were considered.

Vital signs were categorized as Normal, High, or Low based using the Pediatric and Basic Life Support (PALS) age-based criteria for normal vital signs in children^22,23^ and normal thresholds in adults. Missing vital signs data were handled by creating a nuisance category of “unknown or missing,” to indicate that such vital signs were not recorded or were missing in the database. Patients who were on mechanical ventilators or who died were classified as having had severe COVID-19. Patients on mechanical ventilators were identified using the Current Procedure Terminology, Version 4 (CPT-4) codes as well as by searching the clinical events of all patients for ventilator settings that indicate the use of mechanical ventilation. Patient death was established using the discharge disposition of the patient as recorded in the electronic medical records.

There are several ways to categorize the severity of COVID-19. The goal of this study is to compare the long-term effect of malnutrition on severity of COVID-19. As a result, a severity measure that is both clinically and statistically sound in both children and adults is required. A binary severity measure was selected as severe and mild COVID-19. Severe disease activity was defined as patients placed on a mechanical ventilator or patients who died. This ensures that we are less concern about differences in mortality due to the availability of mechanical ventilators and factors such as severity of illness at the onset of mechanical ventilation and quality of care issues relating to census. Patients who required mechanical ventilation or who died required invasive procedures to reduce morbidity. Other patients were classified as having had mild COVID-19. The outcome variable is therefore “mild” vs “severe” COVID-19.

### Statistical considerations

The generalized variance inflation factor (GVIF) estimates the level of multicollinearity which is the degree correlated variables induce corresponding inflation in variance estimates^24–26^. All qualifying variables were assessed using the GVIF. An a priori decision was made to exclude variables with GVIF of 4 or greater in a stepwise manner.

A mixed effects logistic regression model was developed using random intercepts for hospitals since the data consists of multiple hospitals across the United States^27–29^. The random intercept model was built while controlling for patient demographics, socioeconomic status, first vital signs on admission, and preexisting comorbid conditions. A statistical interaction between age and malnutrition^17^ was introduced to assess if there is an age-dependent association between malnutrition and severe COVID-19. The dataset was divided into 2 to create a model for pediatric patients and another for adult patients using 18 years as the threshold.

### Ethics

This study was approved by the Institutional Review Board of Children’s Hospital of Orange County, Orange, CA 92868 with Institutional Review Board approval number 2008107. The need for informed consent was waived by the Institutional Review Board of Children’s Hospital of Orange County, Orange, CA 92868 and all aspect of the work were carried out in accordance with relevant guidelines/regulations including the Helsinki Declaration.

## Results

Study data consisted of 56 hospitals in the United States, 8,604 pediatric hospitalizations, and 94,495 hospitalizations for adults. The pediatric cohort consisted of patients with a mean age of 6 years and standard deviation of 6 years with 43.1% female, 43.8% male, and 13.1% of unidentified sex. Over half (54.4%) were White, 14.1% Black or African American, 2.1% Asian, and 2.3% American Indian or Alaska Native children. Of the remaining children, 16.6% were of other racial groups and 10.4% of unknown race. Health insurance payer of the children consisted of 33.5% governmental, 40.6% private/commercial, 4.8% self-pay, and 21.1% of other/unknown payer types. Mechanical ventilation was required for 517 of the children and 21 expired with all but 3 having been on a ventilator before death. This resulted in 520 (6.0% of) children being classified as severe COVID-19 patients. A total of 164 (1.9% of) children had a history of malnutrition. Among children with mild COVID-19, 1.5% had a history of malnutrition with incidence significantly increasing to 7.5% among those with severe COVID-19.

Mean age in the adult cohort was 53 years with a standard deviation of 19 years. The population was comprised of 47.7% female, 42.6% male, and 9.7% unidentified sex with 59.3% White, 17.8% Black or African American, 2.6% Asian, 2.2% American Indian or Alaska Native,12.5% of other racial groups, and 5.6% of patients of unknown race. Health insurance payer of the adult patients consisted of 36.7% governmental, 40.8% private/commercial, 9.6% self-pay, and 12.9% of other/unknown payer types. Mechanical ventilation was required for 9,953 adults, 4,706 expired, and 3,236 patients were on mechanical ventilation before death. That is, a large proportion of patients on mechanical ventilation (32.5%) expired. This resulted in 11,423 (12.1% of) adults being classified as severe COVID-19 patients. A total of 2,010 (2.1% of) adult patients had a history of malnutrition. Among adults with mild COVID-19, 1.8% had a history of malnutrition. However, among adults with severe COVID-19, a much higher history of malnutrition (4%) was noted.

Summary statistics by severity of COVID-19 are provided in Table 1 for both children and adults on all variables. There were no issues with multicollinearity as the GVIF were less than 4 for all variables. The results of the corresponding model describing the impact of malnutrition are shown in Table 2. Peripheral results on the variables controlled for in the model are provided in Table 3 for interested readers.

**Table 1:** Summary statistics.

Variable	Levels	COVID-19 Severity, n (%) or mean (sd)			
		Pediatrics*		Adults**	
		Mild	Severe	Mild	Severe
Sex	Female	3517 (43.51)	191 (36.73)	40,881 (49.21)	4228 (37.01)
	Male	3519 (43.53)	252 (48.46)	34,605 (41.66)	5611 (49.12)
	Unknown/missing	1048 (12.96)	77 (14.81)	7586 (9.13)	1584 (13.87)
Age (years)	–	7.76 (6.15)	5.04 (5.99)	51.47 (19.19)	66.29 (16.06)
Race	White	4498 (55.64)	183 (35.19)	49,974 (60.16)	6072 (53.16)
	Black or African American	1132 (14.00)	84 (16.15)	14,390 (17.32)	2455 (21.49)
	Asian or Pacific islander	168 (2.08)	16 (3.08)	2113 (2.54)	361 (3.16)
	American Indian or Alaska Native	195 (2.41)	6 (1.15)	1735 (2.09)	351 (3.07)
	Other racial groups	1314 (16.25)	113 (21.73)	10,253 (12.34)	1543 (13.51)
	Unknown racial group	777 (9.61)	118 (22.69)	4607 (5.55)	641 (5.61)
Payer	Governmental insurance	2728 (33.75)	156 (30.00)	28,795 (34.66)	5911 (51.75)
	Private/commercial insurance	3328 (41.17)	169 (32.50)	35,780 (43.07)	2736 (23.95)
	Self-pay	402 (4.97)	9 (1.73)	8772 (10.56)	324 (2.84)
	Unknown	1626 (20.11)	186 (35.77)	9725 (11.71)	2452 (21.47)
Temperature	Normal	4909 (60.72)	395 (75.96)	47,829 (57.58)	6810 (59.62)
	High	1307 (16.17)	94 (18.08)	9967 (12.00)	2739 (23.98)
	Low	14 (0.17)	10 (1.92)	331 (0.40)	322 (2.82)
	Not assessed/unknown	1854 (22.93)	21 (4.04)	24,945 (30.03)	1552 (13.59)
Heart rate	Normal	2659 (32.89)	295 (56.73)	28,773 (34.64)	5635 (49.33)
	High	1224 (15.14)	193 (37.12)	10,849 (13.06)	4017 (35.17)
	Low	71 (0.88)	12 (2.31)	1362 (1.64)	280 (2.45)
	Not assessed/unknown	4130 (51.09)	20 (3.85)	42,088 (50.66)	1491 (13.05)
Respiratory rate	Normal	5669 (70.13)	264 (50.77)	59,210 (71.28)	4470 (39.13)
	High	1069 (13.22)	195 (37.50)	10,802 (13.00)	5654 (49.50)
	Low	314 (3.88)	40 (7.69)	108 (0.13)	97 (0.85)
	Not assessed/unknown	1032 (12.77)	21 (4.04)	12,952 (15.59)	1202 (10.52)
Systolic blood pressure	Normal	3393 (41.97)	251 (48.27)	24,695 (29.73)	2980 (26.09)
	High	1372 (16.97)	94 (18.08)	38,560 (46.42)	5057 (44.27)
	Low	866 (10.71)	153 (29.42)	7285 (8.77)	2149 (18.81)
	Not assessed/unknown	2453 (30.34)	22 (4.23)	12,532 (15.09)	1237 (10.83)
Diastolic blood pressure	Normal	3529 (43.65)	221 (42.50)	22,962 (27.64)	2430 (21.27)
	High	1242 (15.36)	148 (28.46)	20,987 (25.26)	2351 (20.58)
	Low	861 (10.65)	128 (24.62)	26,572 (31.99)	5398 (47.26)
	Not assessed/unknown	2452 (30.33)	23 (4.42)	12,551 (15.11)	1244 (10.89)
Oxygen saturation	100–95%	6912 (85.50)	355 (68.27)	60,614 (72.97)	4878 (42.70)
	94–90%	196 (2.42)	62 (11.92)	8248 (9.93)	2521 (22.07)
	< 90%	88 (1.09)	84 (16.15)	2161 (2.60)	2809 (24.59)
	Not assessed/unknown	888 (10.98)	19 (3.65)	12,049 (14.50)	1215 (10.64)
Malnutrition (E40–E46)	No	7959 (98.45)	481 (92.50)	81,515 (98.13)	10,970 (96.03)
	Yes	125 (1.55)	39 (7.50)	1557 (1.87)	453 (3.97)
**Comorbidities**					
Endocrine, other nutritional, and metabolic conditions (E00-E39, E47-E89)	No	7260 (89.81)	382 (73.46)	54,763 (65.92)	5975 (52.31)
	Yes	824 (10.19)	138 (26.54)	28,309 (34.08)	5448 (47.69)
Nervous system disorders (G00-G99)	No	7463 (92.32)	389 (74.81)	65,743 (79.14)	8049 (70.46)
	Yes	621 (7.68)	131 (25.19)	17,329 (20.86)	3374 (29.54)
Circulatory system disorders (I00-I99)	No	7622 (94.29)	378 (72.69)	55,653 (66.99)	5717 (50.05)
	Yes	462 (5.71)	142 (27.31)	27,419 (33.01)	5706 (49.95)
Respiratory system conditions (J00–J99)	No	5441 (67.31)	335 (64.42)	58,157 (70.01)	7650 (66.97)
	Yes	2643 (32.69)	185 (35.58)	24,915 (29.99)	3773 (33.03)
Congenital malformations, deformations and chromosomal abnormalities (Q00–Q99)	No	7307 (90.39)	358 (68.85)	81,295 (97.86)	11,100 (97.17)
	Yes	777 (9.61)	162 (31.15)	1777 (2.14)	323 (2.83)

*All *p* values are less than 0.0001 except patient sex (0.0101) and respiratory comorbidities (0.1908).

**All *p* values are less than 0.0001 without exception.

**Table 2:** Statistical interaction between history of malnutrition and severe COVID-19 extracted from the full multivariable model.

Variables	Levels	Pediatrics		Adults	
		Log odds ratio (95% CI)	*p* values	Log odds ratio (95% CI)	*p* values
Age	–	− 0.099 (− 0.121, − 0.076)	< 0.0001	0.030 (0.028, 0.031)	< 0.0001
Malnutrition	Yes	− 0.452 (− 1.230, 0.326)	0.2549	1.077 (0.558, 1.596)	< 0.0001
Malnutrition-age interaction	–	0.094 (0.012, 0.175)	0.0245	− 0.014 (− 0.021, − 0.006)	0.0003

**Table 3:** Peripheral results on variables controlled for in the random intercept model for malnutrition.

Variables	Levels	Pediatrics		Adults	
		Odds ratio (95% CI)	*p* values	Odds ratio (95% CI)	*p* values
Sex	Female	Reference			
	Male	1.078 (0.857, 1.358)	0.5201	1.487 (1.415, 1.563)	< 0.0001
	Unknown	0.99 (0.713, 1.375)	0.9512	1.341 (1.24, 1.451)	< 0.0001
Race	White	Reference			
	Black or African American	1.494 (1.07, 2.086)	0.0183	1.186 (1.109, 1.268)	< 0.0001
	Asian or Pacific islander	1.984 (1.077, 3.654)	0.0279	1.143 (0.998, 1.31)	0.0539
	American Indian or Alaska Native	2.51 (0.707, 8.906)	0.1545	1.903 (1.532, 2.362)	< 0.0001
	Other racial groups	1.255 (0.917, 1.717)	0.1565	1.114 (1.03, 1.204)	0.0071
	Unknown racial group	2.619 (1.912, 3.588)	< 0.0001	1.32 (1.18, 1.476)	< 0.0001
Payer	Governmental insurance	Reference			
	Private/commercial insurance	0.91 (0.663, 1.25)	0.5602	0.858 (0.806, 0.913)	< 0.0001
	Self-pay	0.703 (0.317, 1.56)	0.3867	0.565 (0.496, 0.643)	< 0.0001
	Unknown	0.808 (0.553, 1.18)	0.2694	1.199 (1.105, 1.301)	< 0.0001
Temperature	Normal	Reference			
	High	0.853 (0.64, 1.136)	0.2755	1.125 (1.06, 1.194)	0.0001
	Low	4.511 (1.478, 13.771)	0.0081	3.732 (3.099, 4.493)	< 0.0001
Heart rate	Normal	Reference			
	High	1.731 (1.332, 2.249)	< 0.0001	1.665 (1.576, 1.76)	< 0.0001
	Low	1.397 (0.662, 2.949)	0.3797	0.959 (0.826, 1.113)	0.5789
Respiratory rate	Normal	Reference			
	High	1.928 (1.503, 2.473)	< 0.0001	2.8 (2.657, 2.95)	< 0.0001
	Low	2.06 (1.317, 3.222)	0.0015	6.912 (4.942, 9.669)	< 0.0001
Systolic blood pressure	Normal	Reference			
	High	0.698 (0.516, 0.944)	0.0195	0.982 (0.924, 1.044)	0.5597
	Low	1.984 (1.478, 2.663)	< 0.0001	1.42 (1.317, 1.532)	< 0.0001
Diastolic blood pressure	Normal	Reference			
	High	1.406 (1.062, 1.86)	0.0173	1.005 (0.935, 1.079)	0.9013
	Low	2.227 (1.628, 3.045)	< 0.001	1.276 (1.196, 1.361)	< 0.0001
Oxygen saturation	100–95%	Reference			
	94–90%	3.416 (2.35, 4.966)	< 0.0001	1.723 (1.623, 1.83)	< 0.0001
	< 90%	9.841 (6.642, 14.581)	< 0.0001	5.55 (5.164, 5.965)	< 0.0001
Endocrine, other nutritional, and metabolic conditions	Yes	1.215 (0.842, 1.752)	0.2974	0.975 (0.908, 1.047)	0.4918
Nervous system disorders	Yes	1.723 (1.235, 2.403)	0.0014	1.066 (1.002, 1.134)	0.0440
Circulatory system disorders	Yes	2.361 (1.651, 3.377)	< 0.0001	1.078 (1.003, 1.159)	0.0425
Respiratory system conditions	Yes	0.636 (0.473, 0.856)	0.0028	0.93 (0.876, 0.987)	0.0168
Congenital malformations, deformations and chromosomal abnormalities	Yes	1.574 (1.154, 2.145)	0.0041	1.068 (0.927, 1.23)	0.3617

**Figure 1:**
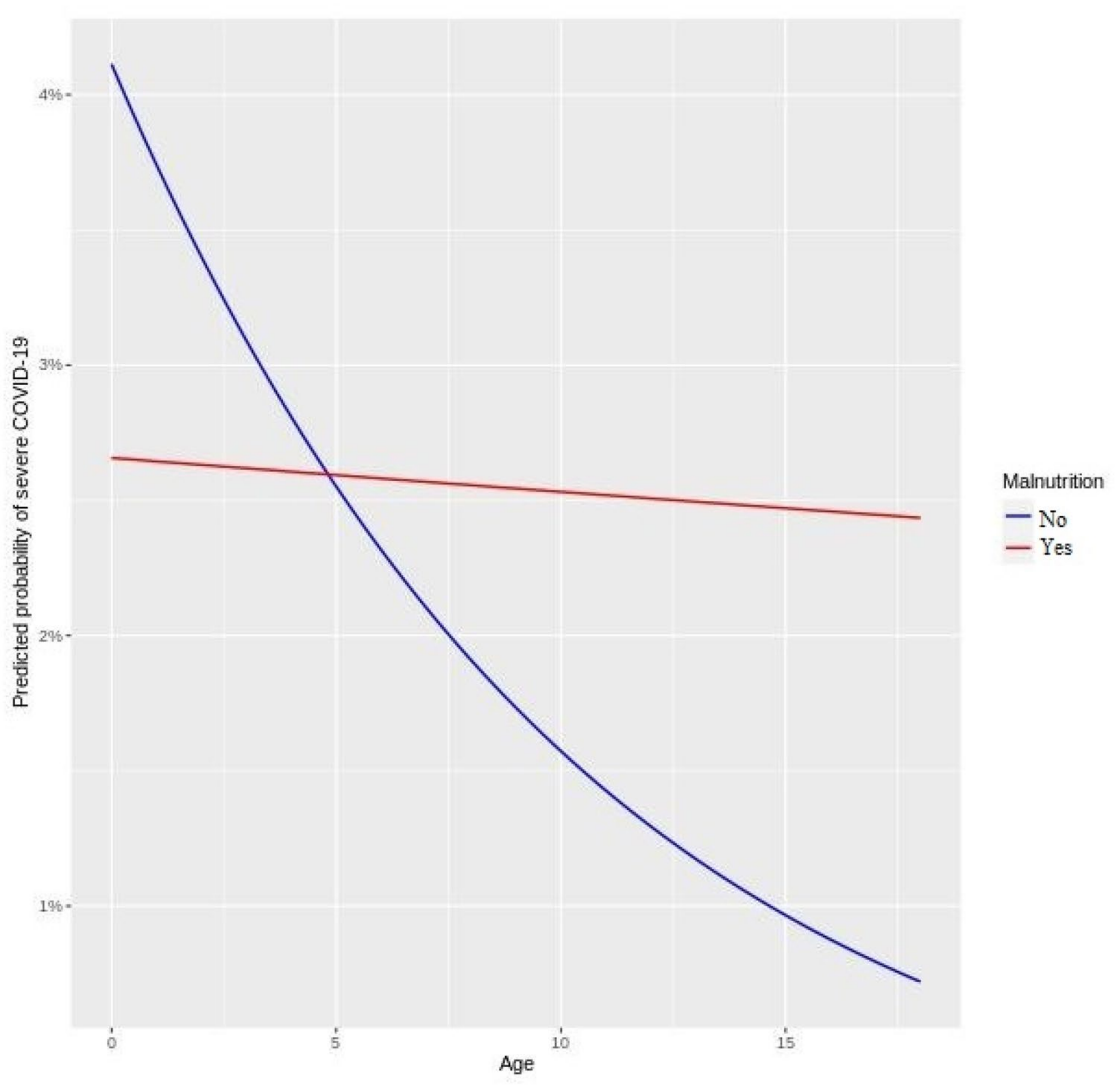
COVID-19 severity and interaction between malnutrition and age in pediatrics.

**Figure 2:**
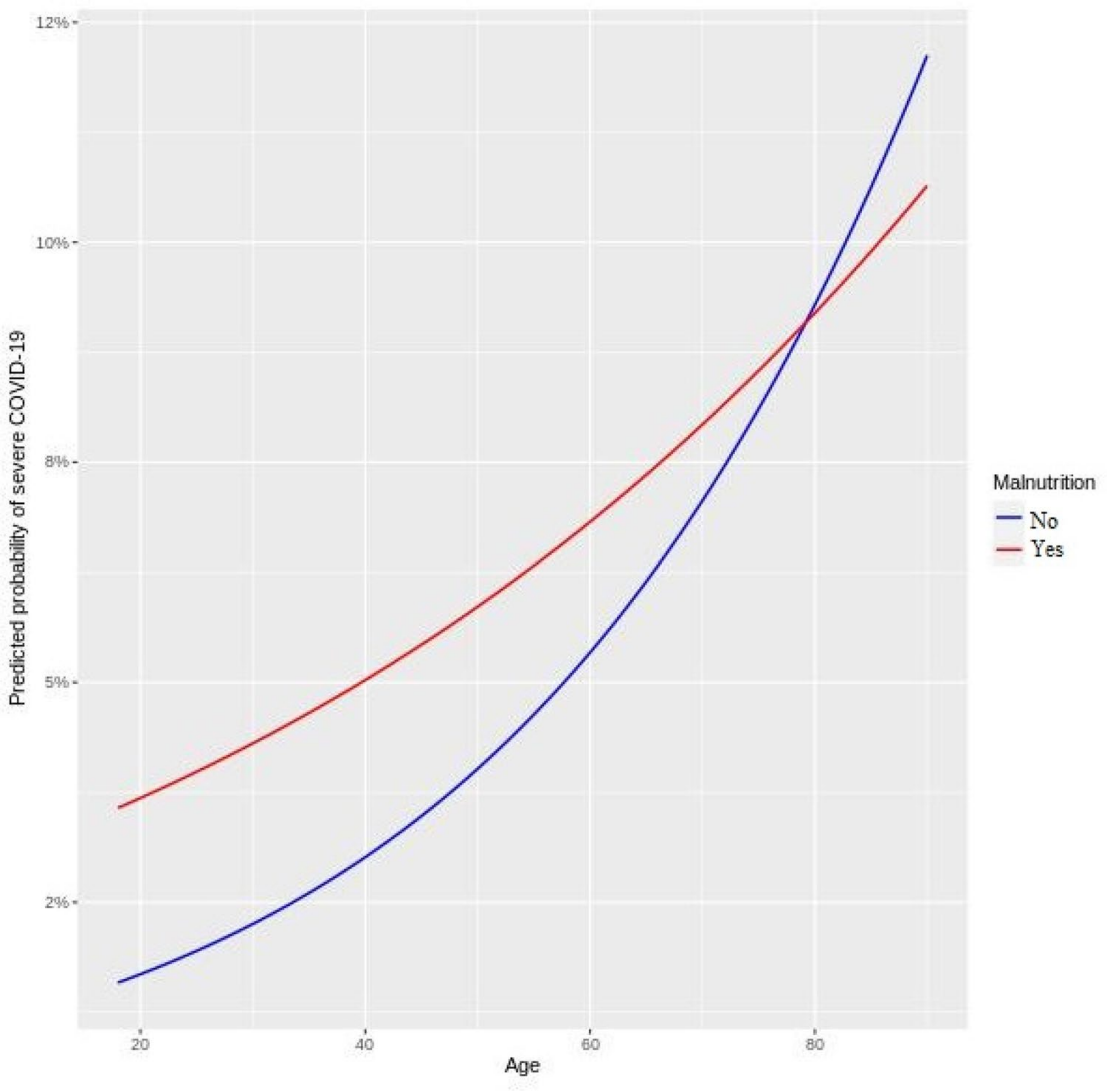
COVID-19 severity and interaction between malnutrition and age in adults.

The multivariable random intercept logistic regression model (accounting for heterogeneity across hospitals) indicated that malnutrition impacts the predisposition of both children and adults to severe COVID-19 through a statistical interaction with age. In other words, the statistical interaction terms in both the pediatric (*p* value: 0.0245) and adult (*p* value: 0.0003) models were significant. This significance implies that the extent of the risk of severe COVID-19 due to malnutrition is dependent on the age of the patient. The main effect terms (in log-odds) for age and malnutrition as well as the size and direction of the interaction terms (also in log-odds) are shown in Table 2. Graphical interpretation of the interactions between malnutrition and patient age are shown in Figs. 1 and 2.

The predicted probabilities were estimated by setting the values of other variables (controlled for in the model) at the most frequent level of each categorical variable. In Fig. 1, the interaction between malnutrition and the age of children indicates the following. First, among malnourished children (children with history of malnutrition), those less than 5 years have the highest odds of severe COVID-19 with slightly lower odds for teenagers. There was a decrease in odds with increasing age in both groups. Second, among patients with no history of malnutrition, younger children less than 5 years were also at higher odds than their teenage peers with a steeper slope. Lastly, malnourished children older than 5 years are at higher odds of severe COVID-19 than their peers with no history of malnutrition. A crossover effect occurred at 5 years in the pediatric model—this may be artifact of having shorter than 5 years medical history on patients less than 5 years in the model.

In Fig. 2, the interaction between malnutrition and patient age in adults indicates the following. The odds of severe COVID-19 increased among malnourished adults (adults with history of malnutrition) between 18 and 78 years and at higher values than their peers with no history of malnutrition. A crossover effect took place at 79 years wherein patients with no history of malnutrition had higher odds of severe COVID-19 than their malnourished peers although the risk of both groups continued to rise with age.

## Discussion

There is an association between past diagnoses of malnutrition and severe COVID-19 through a statistical interaction with the age of the patient in both pediatric and adult medicine. On the one hand, in the pediatric population, the effect of a history of malnutrition on severe COVID-19 is highest in younger children (less than 5 years) but this risk drops only slightly with age. On the other hand, children who are not at risk of malnutrition (or who have no history of a diagnosis for malnutrition) have a much steeper drop in risk for severe COVID-19 with age, such that patients over 5 years of age are at lower risk for severe COVID-19 than their peers with malnutrition. Consequently, all children at risk of malnutrition require nutritional care and support to address their nutritional needs^4^. However, the most opportunity for reducing the risk of severe COVID-19 through nutritional intervention exists among older children.

Poor outcomes due to COVID-19 are more dire among older adults. This study established that there is a larger gap in COVID-19 outcomes among adults between 18 and 78 years than in older patients. These imply that there is an opportunity to target and improve the outcomes of younger adults with nutritional issues although older patients are more likely to have worse outcomes in general. It is already well known that nutritional assessments play a significant role in the clinical course of patients at all ages. But understanding the gaps in COVID-19 outcomes attributable to malnutrition provides an opportunity to improve the prognosis of patients with history of malnutrition.

Malnutrition, as a global health problem for both the pediatric and adult population, will continue to overlap with the COVID-19 pandemic that has already affected millions worldwide^5,6,10,11^. It is imperative to target communities at highest risk of both malnutrition and COVID-19 as COVID-19 has been shown to increase the incidence of malnutrition^10^ and malnutrition is associated with more severe disease in patients of certain age groups. These communities may very well be overlapping as malnutrition and COVID-19 have been seen to disproportionately affect communities that are most vulnerable to health disparities^30,31^. Another opportunity for improving the quality of care of at-risk community (such as those suffering disproportionately from malnutrition) is education to ensure that these patients take advantage of resources addressing food insecurity and do not delay care on developing symptoms of COVID-19 as such delays may further worsen disease outcomes^32,33^. This deeper understanding of the interplay between malnutrition and COVID-19 disease severity underlines the importance of nutritional assessment and intervention across all patient populations, particularly those at risk for COVID-19.

There were several limitations of this study. History of malnutrition was determined using diagnosis codes which may underestimate its prevalence. Furthermore, all diagnosis histories were determined by searching patient records between 2015 and 2019 indicating that patients less than 5 years would have less data available. This may have an impact on the crossover effect in pediatrics observed at 5 years for pediatrics in the interaction between malnutrition and age. However, diagnosis codes remain the most reliable and potentially consistent estimate of malnutrition that cuts across both pediatric and adult medicine in a multicenter study. There was no way to fully establish the primary cause of death or use of mechanical ventilation in this large multicenter study.

We grouped severe, moderate, and mild malnutrition to avoid statistical problems with small sample sizes especially when considering 2-way statistical interactions. While the degree of malnutrition is identified as part of nutritional assessments, we chose to group all levels of malnutrition into a single category to estimate average effect across all severity levels. We were, therefore, able to assess the average effect of malnutrition as a first step with the reasonable assumption that any effect found would be worse in patients with severe malnutrition. Lastly, we were unable to distinguish between chronic and acute malnutrition related to acute illness.

There are several and richer methods for classifying severity of COVID-19. We chose a binary outcome to simplify the analyses given the need to assess statistical interactions^17^. First, we needed an outcome that can be studied in both children and adult. The low number of deaths in children makes it statistically infeasible to separate children who died. Furthermore, there are several factors associated with survival of mechanical ventilation independent of COVID-19. These factors (such as severity of illness at the onset of mechanical ventilation) are exacerbated due to potential shortage of ventilators or unusually high census in the hardest hit areas of the country. A simplification, therefore, was to focus on the need of mechanical ventilation for survival encompassing deaths that may occur before a patient can be placed on invasive mechanical ventilation. Furthermore, a binary outcome reduces the complexity of the statistical model especially given the need for assessment of statistical interactions^17^.

Emphasis has been placed very early into the pandemic on the impact of age and comorbidities on the risk of severe COVID-19 and obesity has been the focus of the nutrition conversation. This study established that the long-term effect of (or preexisting) malnutrition is also a critically important piece of the puzzle. While the COVID-19 pandemic may lead to increased incidence of malnutrition, malnourished patients or patients at risk of malnutrition are also at risk of suffering more severe forms of the disease. Preexisting disparities and new disparities created by COVID-19 may increase health care risks. It is therefore critical that additional studies are carried out and that public health policies affecting patients most at risk for COVID-19 and malnutrition be carefully weighed, in what may be a double-edged sword for patients at risk of malnutrition.

## Data Availability

The dataset analyzed in this study are available from the corresponding authors on reasonable request and upon approval by the Institutional Review Board (IRB) of the corresponding authors’ institution to share the data.
